# IL‐10 differentially controls the infiltration of inflammatory macrophages and antigen‐presenting cells during inflammation

**DOI:** 10.1002/eji.201646528

**Published:** 2016-07-28

**Authors:** Chia‐Te Liao, Marcela Rosas, Luke C. Davies, Peter J. Giles, Victoria J. Tyrrell, Valerie B. O'Donnell, Nicholas Topley, Ian R. Humphreys, Donald J. Fraser, Simon A. Jones, Philip R. Taylor

**Affiliations:** ^1^ Division of Infection and Immunity, Cardiff University School of MedicineHeath ParkCardiffUK; ^2^ Central Biotechnology Services, Cardiff University School of MedicineHeath ParkCardiffUK; ^3^ Systems Immunity University Research Institute, Cardiff University School of MedicineHeath ParkCardiffUK

**Keywords:** Antigen presenting, Antigen processing, Dendritic cells, Fate‐mapping, Inflammation, Macrophages, Monocytes

## Abstract

The inflammatory activation and recruitment of defined myeloid populations is essential for controlling the bridge between innate and adaptive immunity and shaping the immune response to microbial challenge. However, these cells exhibit significant functional heterogeneity and the inflammatory signals that differentially influence their effector characteristics are poorly characterized. In this study, we defined the phenotype of discrete subsets of effective antigen‐presenting cells (APCs) in the peritoneal cavity during peritonitis. When the functional properties of these cells were compared to inflammatory monocyte‐derived macrophages we noted differential responses to the immune‐modulatory cytokine IL‐10. In contrast to the suppressive actions of IL‐10 on inflammatory macrophages, the recruitment of APCs was relatively refractory and we found no evidence for selective inhibition of APC differentiation. This differential response of myeloid cell subsets to IL‐10 may thus have limited impact on development of potentially tissue‐damaging adaptive immune responses, while restricting the magnitude of the inflammatory response. These findings may have clinical relevance in the context of peritoneal dialysis patients, where recurrent infections are associated with immune‐mediated membrane dysfunction, treatment failure, and increased morbidity.

## Introduction

Local immunity in the peritoneal cavity contributes to the failure of peritoneal dialysis (PD) as a treatment for end‐stage kidney disease. In this context, peritoneal infection is a major driver, together with inflammation‐driven fibrosis that leads to peritoneal membrane dysfunction and, in a minority of cases, a severe encapsulating peritoneal sclerosis 1, 2, 3. Positive outcome of infectious peritonitis in PD patients is associated with Th1‐like responses 4 and we recently demonstrated a role for IL‐6/IFNγ/STAT1‐dependent adaptive immunity in the development of fibrotic tissue damage following recurrent peritonitis 5. However, limited information is currently available on the innate sensing cells that control these downstream adaptive immune responses and control of local immune responses could feasibly alleviate tissue damage.

Peripheral tissues contain discrete populations of tissue resident (Res) macrophages (MØ) and dendritic cells (DC), which play fundamental roles in tissue homeostasis and immune‐surveillance 6, 7, 8, 9. In spite of the importance of peritoneal immune alterations in the efficacy of PD and potentially in other conditions, peritoneal antigen presenting cells (APCs) are very poorly characterized 10. In addition, intense historical study of ‘peritoneal MØ’ has largely neglected the phenotypic heterogeneity of these cells, with potential ramifications for understanding many aspects of basic immune‐biology of the tissue, such as which are the key antigen presenting cells within the tissue.

We previously demonstrated in the peritoneum of naïve mice, cells with an F4/80^int‐low^CD11c^int‐low^MHCII^high^CD116^high^CD206^high^12/15‐lipoxygenase^–^ phenotype 11, 12. We tentatively classified these cells ‘DC‐like’ because they mounted effective inflammatory responses 11, 13, presented antigen to naïve CD4^+^ T cells, and were efficient IL‐12 producers. However, during experimental peritonitis we were unable to distinguish the ‘DC‐like’ cells from inflammatory monocyte‐derived (Inf) MØ 11. The presence of these MHCII^high^ ‘DC/MØ‐like’ cells has been confirmed 14, 15, however, critically, the failure to identify them in inflammation has led to ambiguity, and confusion of these cells with InfMØ and possibly also with DC 15, 16.

We now define specific peritoneal MHCII^high^ ‘DC/MØ‐like’ subsets and distinguish them from InfMØ. Contrary to recent suggestions 15, the recruitment of these cells was more resistant to the anti‐inflammatory actions of IL‐10 than contemporary InfMØ. Collectively, our data indicate that effective antigen processing and presentation is restricted to discrete subsets of peripherally‐recruited monocyte‐like cells and identifies differential control of MØ and APC recruitment by IL‐10, which has marked implications for understanding the peritoneal immunity and the tissue damaging adaptive responses that evolve during peritoneal dialysis.

## Results

### Identification of peritoneal APC subsets

**Figure 1:**
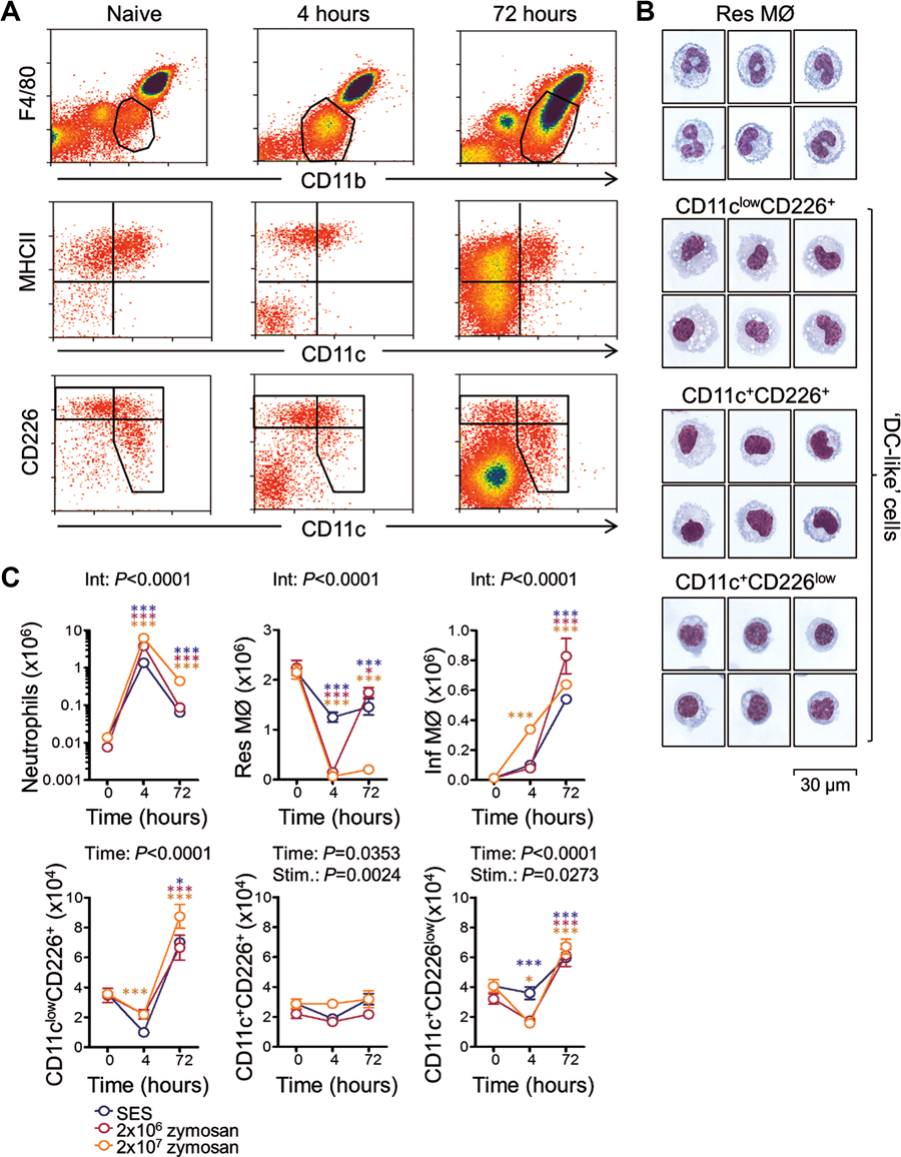
Identification and inflammatory kinetics of peritoneal APCs. (A) Peritonitis was induced by intraperitoneal administration of cell‐free supernatant prepared from a clinical isolate of *S. epidermidis*. Flow‐cytometric analysis showing the kinetic changes of ResMØ and APC during SES‐induced peritonitis 18. Cells were first gated on single cell events (via FSCArea vs FSClinear profiles) and then the APC were gated on via their characteristic F4/80, CD11b profiles (shown in the upper row). The middle and lower rows show comparisons of MHCII and CD226 respectively to CD11c (B cells were confirmed to be excluded via CD19 staining). Last, the APC were subdivided with the aid of CD226 and CD11c expression (i.e. they were CD19^−^CD11b^int^MHCII^+^ with CD11c^low/−^CD226^+^, CD11c^+^CD226^+^, or CD11c^+^CD226^low/−^ phenotypes). Dot plots are from representative mice from one of two independent experiments with 9–11 mice per experiment (B) ResMØ (CD19^−^CD11b^high^MHCII^−^CD11c^low/−^CD226^−^) and the three arbitrary subsets of APC defined above were purified based on these phenotypes (see methods for details) from cells pooled from 10 naïve C57BL/6 mice. The morphology of individual cell subsets on cytospin preparations is shown (scale bar represents 30 μm). Data are representative of two independent experiments. (C) Graphs showing the absolute numbers of neutrophils (F4/80^−^ Ly6G^+^), InfMØ (F4/80^int^CD11b^int^CD11c^−^CD226^−^), ResMØ and the three subsets of APC (as defined above) during acute peritoneal inflammation induced with SES (blue), 2 × 10^6^ (magenta) or 2 × 10^7^ (orange) zymosan particles. Data (mean ± SEM) are derived from 5 to 6 week old C57BL/6 mice (*n* = 3–5 per group). Data from the SES model are derived from one of two independent experiments (9 and 11 mice in total), while data from the zymosan peritonitis experiments are derived from single experiments with a total of 12 and 13 mice. Data were analyzed by two‐way ANOVA (after log transformation in the case of neutrophils) with Bonferroni posttests (color coded *p* value abbreviations relate to differences from time 0 h; *p* values are summarized as follows: *p < 0.05 and ^***^ *p* < 0.001). The interaction statistic (Int) is indicated where significant; else the significance tests for time or inflammatory stimulus (Stim.) are shown.

Myeloid cells in the peritoneal cavity are differentially characterized by expression of CD11b and F4/80 11, 14. We previously considered a minor population with CD11b^int^F4/80^int‐low^ MHCII^high^CD11c^int‐low^ phenotype ‘DC‐like’ because of their ability to present antigen to naïve T cells 11. To discriminate these cells from monocyte‐derived InfMØ during inflammation, published microarray data that compared peritoneal ‘F4/80^low^MHCII^+^’ (a most‐likely mixed population containing the peritoneal APC) with ‘F4/80^int^MHCII^+^’ InfMØ during thioglycollate‐induced peritonitis were analyzed for differences (Supporting Information Table 1) 17. F4/80^low^MHCII^+^ cells were associated with higher expression of CD226 (DNAM‐1, DNAX accessory protein‐1) (Fig. 1A). Polychromatic flow‐cytometric analysis failed to identify a discrete subset of cells, but the majority of cells exhibited moderate or greater staining for CD11c and/or CD226 and could be readily identified throughout an acute inflammatory episode (Fig. 1A). ResMØ and putative APC were purified from naïve animals (Fig. 1B, Methods). All cells were morphologically myeloid, however, the CD11c^+^CD226^low^ cells were smaller in size with less cytoplasm.

### APC kinetics during SES‐induced peritonitis

In the acute *Staphylococcus epidermidis* cell‐free supernatant (SES)‐induced and zymosan‐induced sterile peritonitis models 18, 19, 20, neutrophils (CD11b^+^F4/80^–^Ly6G^+^) are rapidly recruited into the peritoneal cavity during the first few hours, but numbers subside after 24 h 18, 19, 20 (Fig. 1C). In contrast, the numbers of ResMØ decline initially and recover during the resolution of inflammation 19, 20 (Fig. 1C). InfMØ (CD11b^int^F4/80^int^CD11c^–^CD226^–^, Fig. 1A) accumulated in the peritoneal cavity after SES or zymosan administration (Fig. 1C). Examination of the APC subsets (CD11c^low^CD226^+^, CD11c^+^CD226^+^ and CD11c^+^CD226^low^ cells) during the inflammatory response (Fig. 1C), showed the numbers of CD11c^low^CD226^+^ and CD11c^+^CD226^low^ cells initially decreased following peritonitis and then increased later during inflammation (Fig. 1C).

### Origin of peritoneal antigen presenting cells

To determine whether the increased CD11c^low^CD226^+^ and CD11c^+^CD226^low^ numbers were due to the recruitment from the periphery or local expansion, adoptive transfer experiments (Supporting Information Fig. 1) and proliferation studies (Supporting Information Fig. 2) were performed. These indicated that, in contrast to donor ResMØ, which remained relatively stable within the tissue 20, the donor APC were lost from the tissue and replaced by host cells. This result also reflected the relatively poor capacity of the CD226 and CD11c expressing APC subsets to proliferate during inflammation (Supporting Information Fig. 2), in contrast to the recently described proliferative activity of ResMØ 19, 20.

### Dendritic cell precursor fate‐mapping and growth factor dependency of peritoneal APCs

**Figure 2:**
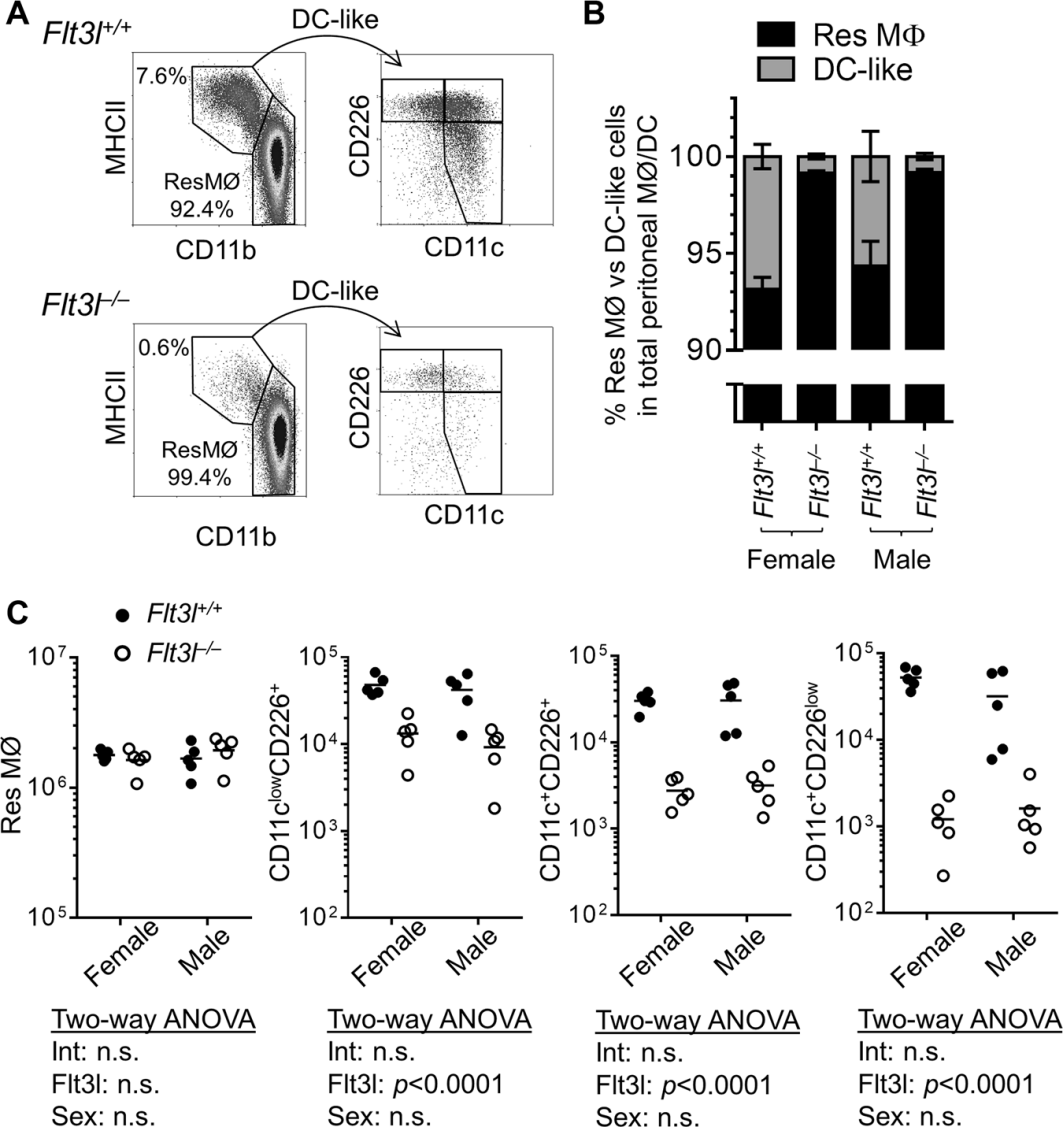
Flt3 ligand dependency of peritoneal APCs. (A) The numbers of peritoneal CD11b^+^MHCII^high^ DC‐like cells from Flt3l‐deficient mice were analyzed by flow cytometry. Density plots are pregated on single cell CD11b^+^F4/80^low/+^ events, and are from an experiment with 20 mice (5 mice per group). (B**)** The relative defect in APC numbers was quantified and expressed as a proportion of CD11b^+^ cells (including ResMØ) (data shown is mean±SEM are derived from an experiment with 5 mice per group). (C**)** Absolute numbers of each myeloid subset in the same mice shown in (B) were analyzed by two‐way ANOVA and *p* values are shown for the effects of Flt3l and sex on myeloid subset numbers and for the existence of an interaction (Int) between these two variables. n.s. denotes ‘not significant’.

**Figure 3:**
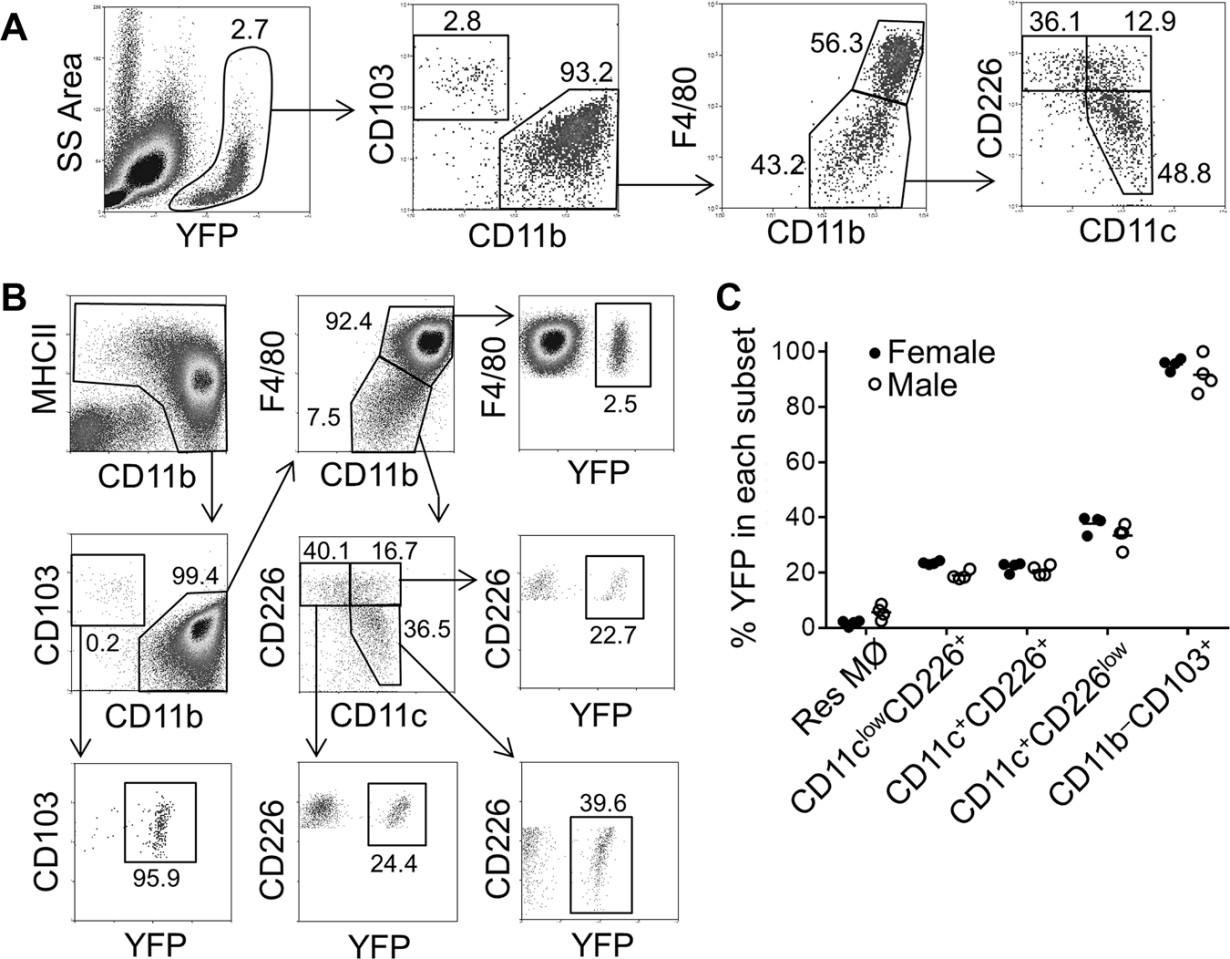
*Clec9a^+/Cre^Rosa^+/EYFP^* mice define DC‐restricted YFP expression in the peritoneal APC. Cells from the peritoneal cavity of *Clec9a^+/Cre^Rosa^+/EYFP^* mice were analyzed for EYFP expression by flow cytometry. A proportion of cells derived from dendritic cell precursors are labeled with EYFP at this precursor stage, and the majority of CD103^+^ DC, which are DNGR‐1^+^, are similarly labeled. (A**)** Peritoneal EYFP^+^ cells were analyzed for CD11b, CD103, F4/80, CD11b, CD226, and CD11c expression. Cells were first gated on single cell events, before being sequentially gated as indicated in the plots. Data are derived from an experiment with four male and four female mice. (B) Gating on the various myeloid subsets specifically allowed quantification of the proportion of EYFP‐expressing cells in each compartment (numbers indicate percentages in each region of the annotated density plots). Dot plots in A and B are representative mice from an experiment with eight mice in total (4 male and 4 female) (C) Graphs, which represent the percentage of each cell type that is EYFP positive in the mice shown in (B) above, demonstrate EYFP in a significant proportion of all CD11b^+^ DC‐like cell subsets, but not ResMØ.

An experiment using mice lacking ligand for the receptor fms‐like tyrosine kinase 3 (*Flt3l^−/−^*), which is an important growth factor for DC expansion in the BM, blood and peripheral lymphoid or non‐lymphoid tissues 22, demonstrated a marked deficiency of these peritoneal APC in the absence of Flt3l (Fig. 2). To further define the developmental origins of these peritoneal APC, DNGR‐1‐expressing cells were fate‐mapped using *Clec9a^+/Cre^Rosa^+/EYFP^* mice 21 to trace the DC lineage (common DC precursors and their progeny). Multi‐color flow cytometric analysis delineated the distribution of YFP^+^ DC lineage within the naïve peritoneal cavity (Fig. 3) and the proportion of YFP^+^ cells in individual MØ/DC subset (Fig. 3). Compared to ∼90% YFP^+^ in CD11b^‒^CD103^+^ DC and <5% YFP^+^ in ResMØ, the three APC subsets are around 20–40% YFP^+^ (CD11c^+^CD226^low^ subset is the highest one among them).

### Dependency of peritoneal APCs on CCR2

**Figure 4:**
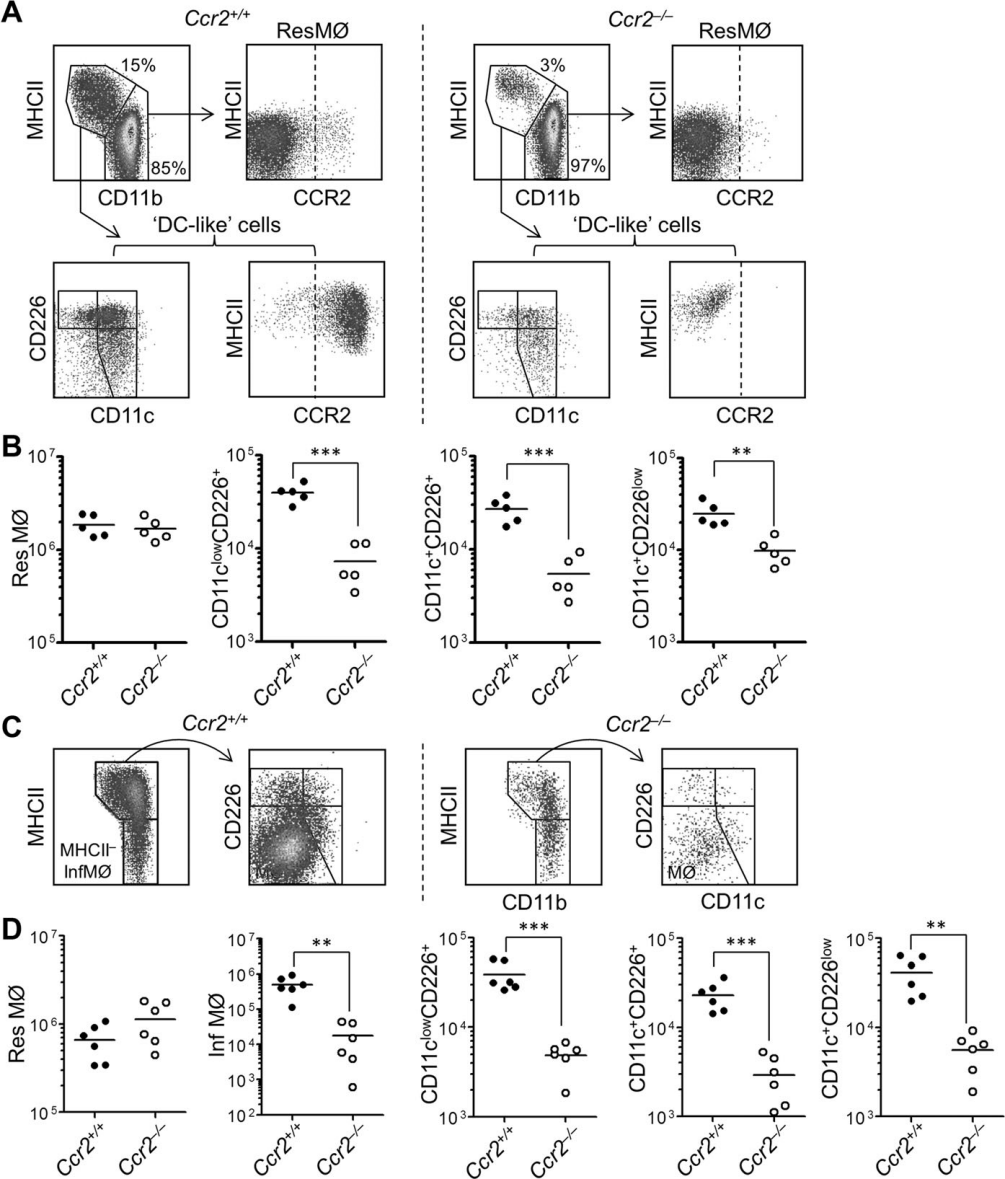
Myeloid cell compositions in the naïve and inflamed peritoneal cavity of *Ccr2^‒/‒^* mice, compared to wild‐type *Ccr2^+/+^* mice. Peritoneal cells from wt and *Ccr2^+/+^* mice were analyzed by flow cytometry. (A) Representative density plots showing peritoneal ResMØ (CD11b^high^MHCII^−^) and three ‘DC‐like’ subsets (CD11b^int^MHCII^+^ with CD11c^low^CD226^+^, CD11c^+^CD226^+^, or CD11c^+^CD226^low^) from naïve C57BL/6 CD45.1 (*Ccr2^+/+^*) mice (left panels) and *Ccr2^‒/‒^* mice (right panels). Myelomonocytic cells (MØs and DC) were pregated as CD19^−^CD11b^+^F4/80^high‐to‐low^ cells. Dashed lines denote the level of isotype controls. In naïve *Ccr2^‒/‒^* mice, the proportion of three ‘DC‐like’ subsets within the MØ/DC pool is decreased. Data are representative of five mice per group from one of two similar experiments. (B) Scatter graphs depicting significantly decreased numbers of all three ‘DC‐like’ subsets in *Ccr2^‒/‒^* mice, compared to *Ccr2^+/+^* mice. Additionally, no differences in total cell number, eosinophils, ResMØ numbers were noted between *Ccr2^+/+^* and *Ccr2* ^‒/‒^ naïve mice under naive status. Horizontal lines represent the mean of five mice per group (Symbols represent individual mice) from one of two independent experiments. Data were analyzed by Student's *t‐*test. (C) Representative density plots showing peritoneal ResMØ, InfMØ, and three ‘DC‐like’ subsets from 72 h after SES challenge in *Ccr2^+/+^* mice (left panels) and *Ccr2^‒/‒^* mice (right panels). Cells were gated as described in (A) above. In *Ccr2^‒/‒^* mice, the proportion/number of CD11b^+^F4/80^int‐to‐low^ InfMØ/DC decreased, compared to *Ccr2^+/+^* mice. Data are derived from one of two similar experiments and represent six mice. (D) Scatter plots graphs depicting significantly decreased numbers of InfMØ and all three ‘DC‐like’ subsets in *Ccr2^‒/‒^* mice, compared to *Ccr2^+/+^* mice, at 72 h after SES challenge. Data represent the mean of six mice *per* group pooled from two independent experiments. Data were analyzed by Student's *t‐*test.

Since the CD226 and CD11c expressing APC uniformly expressed CCR2, their numbers were examined and found to be markedly reduced under both homeostatic and inflammatory conditions in the absence of CCR2 (Fig. 4). The three APC populations were significantly reduced in numbers in naïve mice (Fig. 4A) and also in mice experiencing zymosan peritonitis (Fig. 4B). This was in contrast to the presence of normal numbers of ResMØ in the absence of CCR2 in both naïve and inflamed conditions (Fig. 4), but similar to the deficiency in inflammatory monocyte‐derived MØ observed during inflammation in the absence of CCR2 (Fig. 4B).

### Phenotype and antigen presenting properties of peritoneal APCs

**Figure 5:**
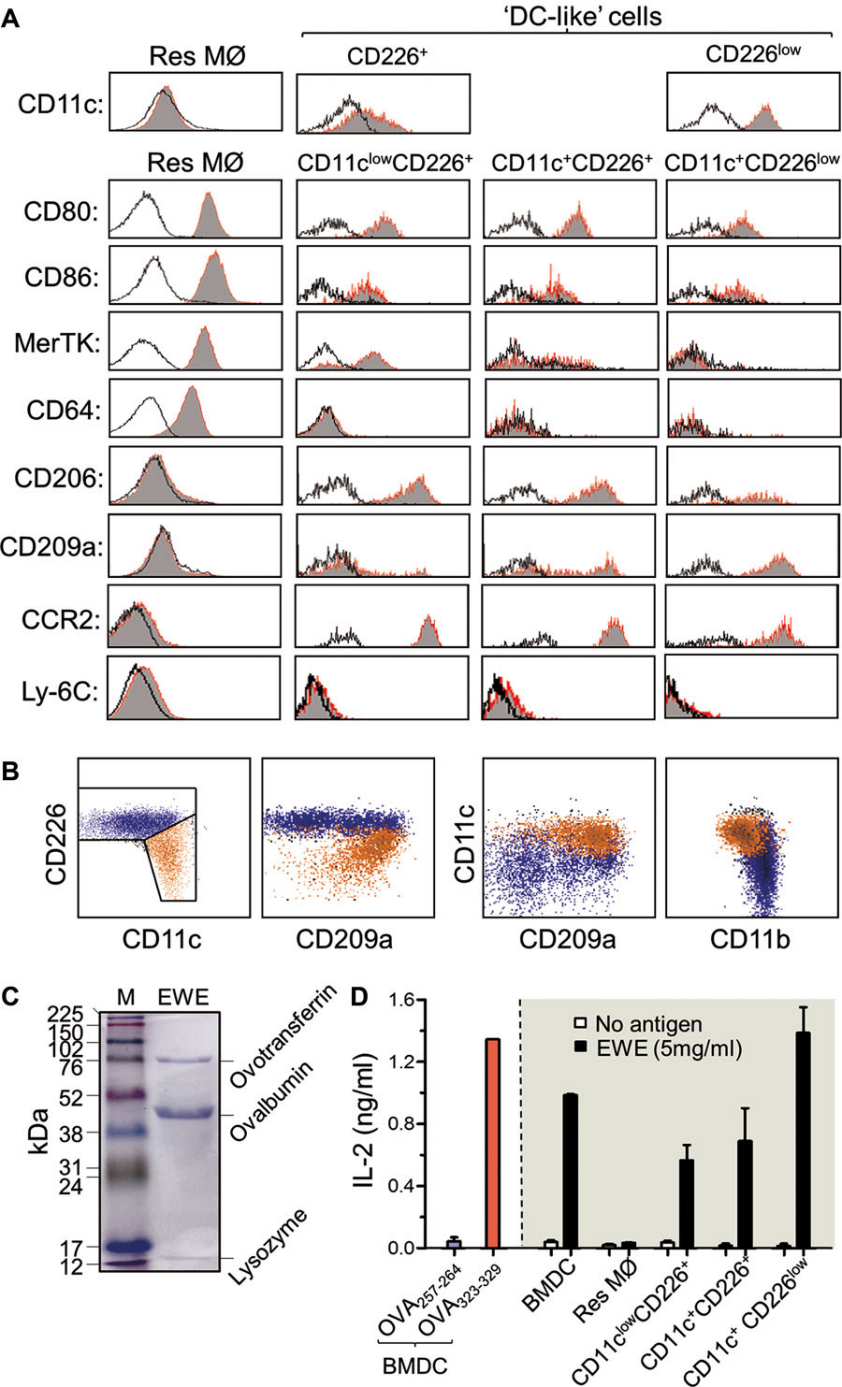
Phenotype and antigen presentation by peritoneal APC in naïve mice. (A) Histograms showing differential expression of surface markers on the defined subsets of peritoneal APC in naïve mice. Data are representative of six mice pooled from two independent experiments with C57BL/6 mice. (B) Multi‐color flow cytometry analysis of peritoneal cells for CD11c, CD209a, and CD226 expression. (C) Coomassie stained SDS PAGE gel showing the protein content of EWE (5 μg resolved on a 12% SDS PAGE gel). Ovalbumin (OVA) (∼45 KDa) is the major constituent of egg white. (D) The ability of the purified cellular subsets to process and present EWE on MHCII to BO‐97.11 cells was assessed by IL‐2 ELISA measurement in the cell culture supernatants. Data show mean±SEM of IL‐2 production by the BO‐97.11 T‐cell hybridoma from two independent experiments, which were pooled after normalization of the data to the peptide pulsed BMDC control.

When first reported, several markers were noted to be differentially expressed between the bulk population of APC and the co‐existing ResMØ in naïve mice (e.g. the mannose receptor CD206) 11. While the CD11c^+^CD226^low^ phenotype appears to define one population of cells, neither CD11c nor CD209a allowed definitive identification of discrete populations (Fig. 5A and B).

Both ResMØ and putative APC express CD80 and CD86 (Fig. 5B), however, only ResMØ expressed both of the proposed ‘MØ‐markers’ CD64 (FcγR1) and MerTK 17 while CD11c^low^CD226^+^ cells from naïve animals did express MerTK (Fig. 5A). Notably, only the putative APC express CD206 (macrophage mannose receptor) 11 and CCR2 (Fig. 5A) in naïve animals. Of the populations, CD11c^+^CD226^low^ cells have more uniformly high CD209a expression (Fig. 5A). A similar expression profile was seen during inflammation (Supporting Information Fig. 3). During inflammation, the CD226^+^ subpopulations exhibited transient Ly‐6C expression (Supporting Information Fig. 4A). A selective expansion of CD226^+^ cells was also observed in mice with a myeloid Gata6‐deficiency 23 (Supporting Information Fig. 4B). Since CD226 high expression was associated with heterogeneous expression of CD11c and CD209a it was pragmatic to continue to divide them by surface antigen phenotype: CD11c^+^CD226^+^, CD11c^low^CD226^+^ and CD11c^+^CD226^low^cells.

### Antigen processing and presentation by peritoneal APCs

To determine if the putative APC were competent antigen presenting cells and to ensure direct measurement of antigen processing and not presentation of unprocessed ‘low quality’ antigen preparations, a fresh egg white extract (EWE) as a source of ovalbumin was prepared 24 (Fig. 5C). Bone marrow (BM)‐derived DC were used live or fixed in antigen presentation assays with EWE or commercial ovalbumin and specific T‐cell hybridomas. Commercial ovalbumin, but not EWE, could be presented by fixed BMDC (Supporting Information Fig. 6), so EWE was used to assess native antigen processing and presentation.

All putative APC processed EWE and presented to BO‐97.11 CD4^+^ T cells, as determined by IL‐2 production. (Fig. 5D). As previously noted 11, the MHCII^low/–^ ResMØ had no significant activity in this assay.

### IL‐10 and the differential regulation of inflammatory MØ and APC recruitment

**Figure 6:**
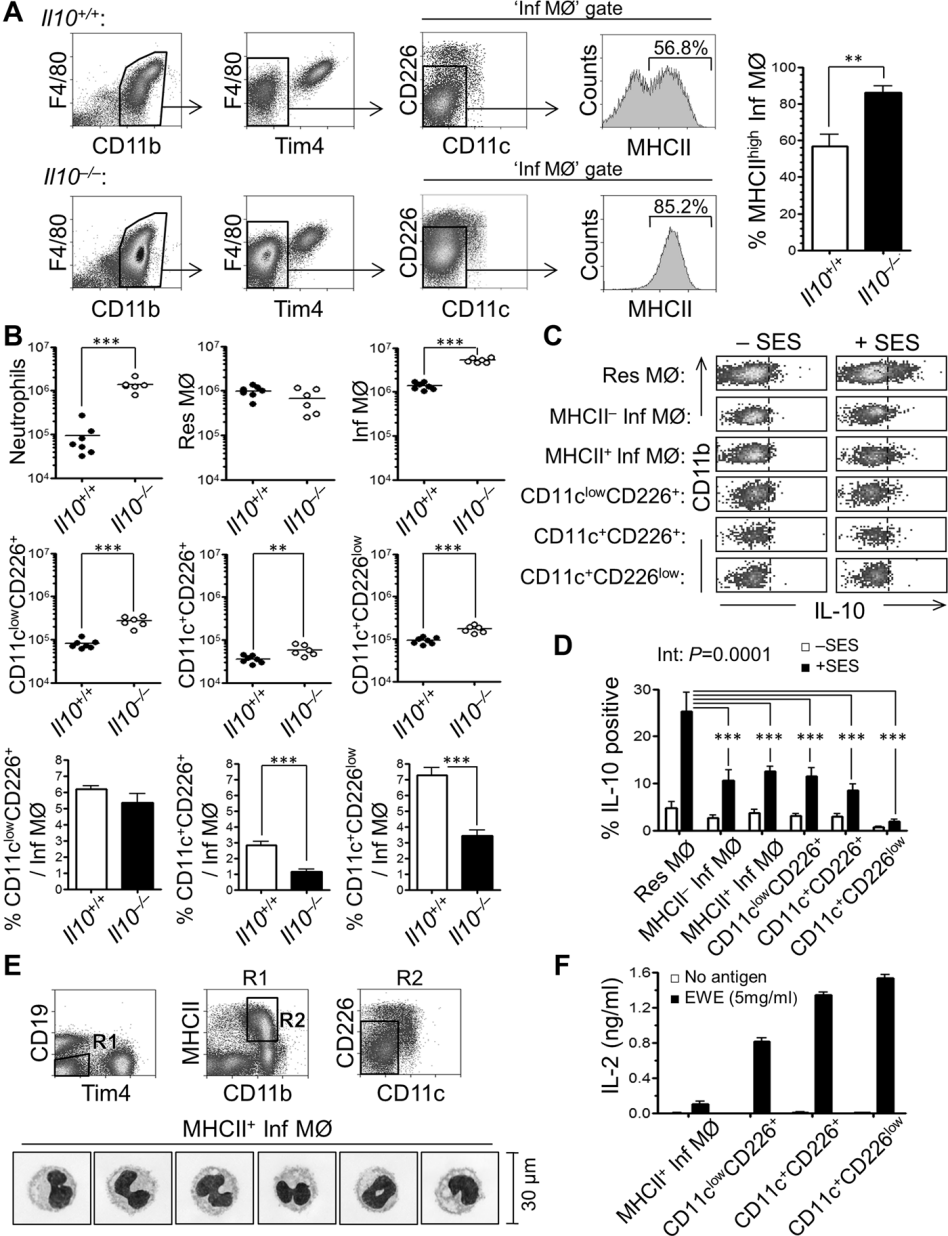
IL‐10 limits the inflammatory response to intraperitoneal SES, but it does not limit the presence of peritoneal APCs. Peritoneal cells from wild‐type and *Il10^−/−^* mice with SES‐induced peritonitis were analyzed by flow cytometry. (A) Density plots showing the gating of recruited InfMØ (CD11b^int^F4/80^int/low^Tim4^−^CD11c^−^CD226^−^) and the proportion of positive MHCII expression at 72 h post‐SES challenge in wild‐type (*Il10^+/+^*, upper panels) and IL‐10 knockout (*Il10^−/−^* lower panels) C57BL/6 mice. Cells were pregated by the exclusion of doublets, F4/80^−^Ly6G^+^ neutrophils, and CD19^+^ B cells. Tim4 antibody was applied to assure the specific exclusion of the majority of ResMØs (CD11b^high^F4/80^high^Tim4^+^). Comparison of high MHCII expression of InfMØs between *Il10^+/+^* and *Il10^−/−^* mice was shown in the bar graph (far right). Data shown are represented as mean±SEM of —six to seven mice per group, combined from two independent similar experiments. Data were analyzed by Student's *t*‐test. (B) Graphs showing the comparisons of actual numbers of individual peritoneal populations between *Il10^+/+^* and *Il10^−/−^* mice at 72‐h post‐SES challenge. Data were derived from same animals shown in (A). *p* values are summarized as follows: ^**^ *p* < 0.01 and ^***^ *p* < 0.001.(C) Representative density plots depicting flow‐cytometric determination of intracellular IL‐10 production from individual subset of peritoneal MØ and APC harvested from C57BL/6 mice 72‐h post‐SES challenge then stimulated with SES (1:10 dilution). (D) Bar graph showing the comparisons of intracellular IL‐10 production among distinct subsets of peritoneal MØ and APC from 72‐h post‐SES challenged C57BL/6 mice. Positive intracellular IL‐10 production was determined by comparison with isotype controls. Data represent the mean±SEM of C57BL/6 female mice from one of two identical experiments with five mice per experiment. Data were analyzed by two‐way ANOVA, with Interaction (Int) statistic indicated and post‐test analysis of the differences from ResMØ (asterisks). *p* values are summarized as follows: ^***^ *p* < 0.001. (E) Dot plots showing the flow cytometric sorting strategy of peritoneal MHCII^+^ InfMØs (CD19^−^Tim4^−^CD11b^+^MHCII^+^CD11c^−^CD226^−^) as well as three subsets of peritoneal APC cells from 72‐h post‐SES challenged C57BL/6 mice. The CD19^–^Tim4^–^ population (R1) was gated, before gating on the CD11b^+^MHC‐II^high/+^ population (R2) and then excluding the three APC subsets (CD226^–^CD11c^–^). The peritoneal APC were purified as previously defined based on CD11c and CD226 expression. The morphology of cytospun MHCII^+^ InfMØ is shown below (scale bar represents 30 μm). Data represent two individual experiments (cells were pooled from 10 C57BL/6 for each experiment). (F) Bar graph depicting the IL‐2 production of BO‐97.11 cells after culture with the indicated cell subsets and EWE. Data represent the mean ± SEM of one of two identical experiments, each with three technical replicates.

IL‐10 has recently been reported to control the differentiation of peritoneal ‘DC‐like’ cells during inflammation from peripheral monocytes 15. This study implied the MHCII^+^ inflammatory cells were related to those found in the naïve tissue, and proposed a role for IL‐10 limiting the differentiation of DC from monocytic precursors. Critically, these APC could not be distinguished from concurrently infiltrating monocyte‐derived InfMØ. Additionally, the presence of the APC populations in naive mice was unaffected by IL‐10‐deficiency (Supporting Information Fig. 7). SES‐induced inflammation in *Il10^–/–^*mice, compared to wild type controls, indicated a marked increase in the influx of neutrophils and InfMØ in the absence of IL‐10 (Fig. 6). Three days after SES injection, InfMØs (CD11b^int^F4/80^int^CD11c^–^CD226^–^) from *Il10^–/–^* mice exhibited more MHCII expression, compared to the *Il10^+/+^* mice (Fig. 6A). Numbers of neutrophils, InfMØs and APC subsets were also significantly increased in *Il10^–/–^* mice compared to the controls (Fig. 6B). To control for the exaggerated inflammatory response observed in the *Il10^–/–^* mice, the numbers of APC subsets were expressed as a ratio to InfMØ. While the numbers of APC were higher in the IL‐10‐deficient mice compared to controls (Fig. 6B, middle row); when considered relative to the InfMØ, the numbers of APC in the knockout mice were lower (Fig. 6B, lower row). Interestingly, ResMØ had the highest propensity to produce IL‐10 upon ex vivo stimulation (Fig. 6C and D), followed by all other MØ‐like cells with minimal IL‐10 production by the CD11c^+^CD226^low^ DC‐like cells.

Given the previous suggestion 15 that the peritoneal APC may share common origins with InfMØ, but knowing that these two cell types could not at that time be distinguished, the potential of MHCII^+^ InfMØ for presentation of native antigen was tested (Fig. 6E and F). MHCII^+^ InfMØ had typical MØ appearance (Fig. 6E), but only a minimal capacity to present ovalbumin to the BO‐97.11 hybridoma (Fig. 6F). Acknowledging the role of CD206 in the uptake of ovalbumin 25 and that the APC exhibited higher CD206 expression than InfMØ (Fig. 5A, Supporting Information Fig. 3), the ability of the MHCII^+^ InfMØ to present the poorly glycosylated 26 hen egg lysozyme antigen to the 2G7 hybridoma (Supporting Information Fig. 8) was tested. These experiments ratified the initial observations of a relatively poor ability of MHCII^+^ InfMØ to process and present antigen, unlike the CD226 and CD11c expressing peritoneal APC subsets.

## Discussion

Peritoneal immune alterations are believed to influence outcome after peritonitis, however, peritoneal APC are very poorly characterized. We recently demonstrated the role of a specific adaptive immune response in the development of fibrotic tissue damage following recurrent peritonitis 5. In this study, our objective was to characterize the local APC as a first step to the definition of the evolution of these local immune responses. We defined a selective surface antigen phenotype for subsets of APC within the peritoneal cavity, permitting the first study of their inflammatory kinetics, activation and capacity for antigen processing and presentation.

While we divided the cells into three populations based on CD226 and CD11c expression for pragmatic reasons, several observations suggest the existence of two main populations of APC in the peritoneum (represented by the CD11c^+^CD226^low^ and CD11c^low^CD226^+^ phenotypes): more evident MerTK expression on the CD226^+^ cells compared to its absence on CD11c^+^ CD226^low^cells (Fig. 5A, Supporting Information Fig. 3); a transient appearance of Ly‐6C expression on the CD226^+^ subpopulations during acute inflammation (Supporting Information Fig. 4A); and a more uniform staining of the CD11c^+^CD226^low^ cells with CD209a (Fig. 5).

We investigated the origins of the peritoneal APC. The development of DC is mostly dependent on Flt3l 27, so we examined the presence of myelomonocytic cells in the peritoneal cavities of Flt3l‐deficient mice (Fig. 2). All three APC populations exhibited a marked dependency on Flt3l as expected of cells of the dendritic cell lineage, but the ResMØ were present in comparable numbers in the Flt3l‐deficient and wild‐type mice. To confirm the presence of DC, we employed DNGR‐1^+^ cell fate mapping using the Clec9a‐Cre to label cells derived from the DC precursors (Fig. 3). Although ResMØ exhibited minimal YFP labeling and CD103^+^CD11b^–^ DC were mostly positive (because of their expression of DNGR‐1), the peritoneal APC exhibit heterogeneous labeling comparable to that seen in bona‐fide DC 21. Collectively, these data strongly support the idea that a substantial fraction of the ‘DC‐like’ cells are products of the DC lineage, as defined by Guilliams *et al*. 27. All three APC populations also expressed CCR2 and, in contrast to ResMØ, they exhibited notable reductions in numbers in the absence of CCR2 (Fig. 4). CCR2‐dependency is generally taken to indicate contribution to the APC populations by the monocyte‐lineage. Using the criteria of Guilliams *et al*. we can further define the nature of the DC population present via marker characterization, with the cells having the phenotype of conventional or classical (c)DC2. This is because of their expression of CD11b, CD206, and CD115 (the latter previously reported 11) and lack of CD103 and Clec9a (the latter evident from the Immunological Genome Consortium data: Immgen.org) 27.

The arrival of the peritoneal APC in the tissue is differentially regulated by IL‐10, as compared to the bulk population of inflammatory monocyte‐derived MØ. This indicates that during inflammation, IL‐10 preferentially suppresses the inflammatory response, with a notably reduced impact on APC recruitment. A recent study of infectious inflammation assessed the regulatory role of IL‐10 on the differentiation of MHCII^+^ ‘DC’ and MHCII^–^ MØ 15. Our data reproduce some observations, showing that IL‐10 regulates MHCII expression by InfMØ during inflammation. However, with regard to IL‐10 preventing differentiation of DC from a common monocytic‐precursor 15, we have shown, the APC subsets are not only proportionately less increased than MHCII^+^ InfMØ in the absence of IL‐10, indicating no preferential restriction of their differentiation, but that their origins are more complex. Collectively, these studies demonstrate that MHCII expression by InfMØ is an indicator of the inflammatory process and the state of their activation. This, along with the recruitment of these cells, is regulated by IL‐10, which may be predominantly derived from ResMØ. However, the presence of efficient peritoneal APC during an acute inflammatory response is more independent of IL‐10. This could indicate that strategies aimed at regulating inflammation through the therapeutic modulation of IL‐10 may have a lesser impact on the development of adaptive immunity, while prominently regulating InfMØ recruitment and activation (MHCII expression). The discrete compartmentalization of APC functions within the tissue has implications for the development of protective immunity at this site and the potential immune‐driven failure of PD as a therapy, particularly in patients who experience repeated bouts of infection 5. Interestingly, like the recruitment of the APC (this study), immune‐driven tissue damage in the peritoneum is less influenced by IL‐10 than inflammatory cell recruitment 5.

## Materials and methods

### Animals

Mice used in this study (C57BL/6, Harlan; C57BL/6.*Il10* ^–/–^, Jackson Laboratory; 129S6/SvEv and 129S6/SvEv.CD45.1 congenic, our colonies; *Ccr2^–/–^* mice: B6.129S4‐*Ccr2* ^tm1lfc^/J, Jackson Laboratory; C57BL/6.CD45.1 (B6.SJL‐*Ptprc* ^a^ *Pepc* ^b^/BoyJ) mice, Jackson Laboratory)) were 6–12 week old females. *Clec9a^+/Cre^Rosa^+/EYFP^* and *Flt3l^‒/‒^* mice, 9–16 week old females and males, were kindly provided by Professor Caetano Reis e Sousa (The Francis Crick Institute, UK). All experiments were performed in accordance with institutional and United Kingdom Home Office guidelines.

Peritonitis was caused by intraperitoneal administration of 200 μL of a cell‐free supernatant prepared from a clinical isolate of *S. epidermidis* (SES) 18 or administration of zymosan particles (0.2–2 × 10^7^, as indicated). Mice were sacrificed and peritoneal cells harvested by lavage using 5 mL ice‐cold 5mM EDTA in PBS.

### Flow‐cytometry

#### Analysis

Flow‐cytometry was performed according to conventional protocols 20, 28, 29, 30, 31. Cells were incubated in blocking buffer (5% (v/v) heat‐inactivated rabbit serum, 0.5% w/v BSA, 5 mM EDTA, 2 mM NaN3, 4 μg/mL rat anti‐mouse FcɣRII&III (2.4G2) in PBS) for 30 min at 4°C. Fluorochrome‐labeled or biotin‐conjugated antibodies (Supporting Information Table 2) in wash buffer (0.5% w/v BSA, 5 mM EDTA, 2 mM NaN_3_ in PBS) were added. After 30‐min incubation at 4°C, cells were washed three times with wash buffer. When necessary, cells were incubated for further 30 min at 4°C with fluorochrome‐conjugated streptavidin (Supporting Information Table S2). For intracellular flow cytometry, cells were fixed in 1% formaldehyde (in PBS) for 20 min and permeabilized with wash buffer containing 0.5% (w/v) saponin (Sigma) prior to staining as above. Cells were acquired on a Cyan ADP analyzer, and analyzed with Summit software (Beckman‐Coulter, Fullerton, CA, USA).

#### Cell sorting

Cells were typically pooled from ∼10 mice (naïve or 72 h after intraperitoneal SES) and purified on a FACSAria^TM^III (BD Bioscience) after staining as above (without NaN_3_). Cell purity was confirmed by flow‐cytometry. Cytospin preparations were made using 10^4^ cells (300 rpm for 5 min, Cytospin 3, Shandon). Slides were stained with Microscopy Hemacolour (Merck), visualized on a Leica DMLB microscope with DFC490 camera (Leica) and processed using QWin Software (Leica). The highest cell sort purities were: ResMØ: 98.56%; MHCII^+^ InfMØ: 99.72%; CD11c^low^CD226^+^: 94.29%; CD11c^+^CD226^+^: 89.86%; CD11c^+^CD226^low^: 88.89%.

### Ex vivo stimulations

Peritoneal cells were washed three times with complete RPMI medium (RPMI 1640 medium supplemented with 10% heat‐inactivated fetal calf serum, 10 U/mL penicillin and 10 μg/mL streptomycin). Cells were stimulated in 48‐well flat‐bottomed plates (2 × 10^5^/well in 200 μL) in the presence of 0.1% (v/v) Golgi‐plug (BD Biosciences). Stimulations with SES (10% (v/v) in complete RPMI) or LPS (100 ng/mL), with medium only controls, were performed in parallel for 6 h (37°C, 5% CO_2_). The cells were carefully recovered using cell scrapers and analyzed (see above).

### APC assays

Bone marrow (BM)‐derived DC from 129S6SvEv mice were differentiated in 20 ng/ml GM‐CSF for 7 days. B3Z, a CD8^+^ T‐cell hybridoma specific for the ovalbumim^257‐264^ peptide (SIINFEKL) in the context of K^b^, was provided by Nilabh Shastri (University of California, Berkeley, CA) 32. BO‐97.11, a CD4^+^ T‐cell hybridoma that responds to the peptide ovalbumin^323‐339^ (ISQAVHAAHAEINEAGR) presented by I‐A^b^, was a gift from Philippa Marrack (National Jewish Center for Respiratory Medicine, Denver, CO, USA) 33. Purified APCs (10^4^ cells) were co‐cultured with BO‐97.11 cells at a 1:1 ratio in a 96‐well flat‐bottom plates in 40μL complete RPMI with or without 5 mg/ml egg white extract (EWE, see below). In control experiments 5 × 10^4^ BMDC, were used in a 1:1 ratio with either B3Z or BO97.11 cells with EWE or ovalbumin. To ensure antigen internalization and processing was occurring, BMDC were fixed with 1% paraformaldehyde for 10 min, which was then quenched with an equal volume of 0.1M glycine in PBS (pH 7.0) for 10 min. APC were washed three times with medium before co‐culture with the T cells for 24 h (37°C, 5% CO_2_). The ovalbumin peptides indicated above (2 μM) were used as controls. IL‐2 in the supernatants was measured by ELISA (BD OptEIA, BD Biosciences).

### EWE

To prepare EWE 24, egg white was recovered through a small hole in the end of a fresh chicken egg and diluted to 50 mL with PBS. The egg white was repeatedly passed through decreasing needle sizes (21G to 25G) and then a 0.2 μm filter before storage at −80°C. A sample of 5 μg was separated on a 12% SDS PAGE gel under reducing conditions and stained with Coomassie blue.

### Statistical analysis

Statistical analyses (see appropriate text) were achieved with GraphPad Prism. *p* values, when summarized, are as follows: *p < 0.05, ^**^ *p* < 0.01, and ^***^ *p* < 0.001.

## Conflict of interest

The authors declare no commercial or financial conflict of interest.

**Abbreviations**
APC: antigen presenting cell
DC: dendritic cell
Inf: inflammatory
MØ: macrophage
PD: peritoneal dialysis
Res: tissue‐resident
SES: *Staphylococcus epidermidis* cell‐free supernatant

## Supporting information

As a service to our authors and readers, this journal provides supporting information supplied by the authors. Such materials are peer reviewed and may be re‐organized for online delivery, but are not copy‐edited or typeset. Technical support issues arising from supporting information (other than missing files) should be addressed to the authors.

Supporting Information
