# Supplementary material for: IL‐10 differentially controls the infiltration of inflammatory macrophages and antigen‐presenting cells during inflammation
Source: Eur J Immunol. 2016 Jul 28;46(9):2222–32. doi: 10.1002/eji.201646528 (PMC5026061; doi:10.1002/eji.201646528)
Supplement: Supplementary file 1 — Supporting Information [file EJI-46-2222-s001.pdf]

European Journal of Immunology

Supporting Information
for
DOI 10.1002/eji.201646528

Chia-Te Liao, Marcela Rosas, Luke C. Davies, Peter J. Giles, Victoria J. Tyrrell, Valerie B. O'Donnell, Nicholas Topley, Ian R. Humphreys, Donald J. Fraser, Simon A. Jones and Philip R. Taylor

IL-10 differentially controls the infiltration of inflammatory macrophages and antigen-presenting cells during inflammation

© 2016 The Authors. *European Journal of Immunology* published by WILEY-VCH Verlag GmbH & Co. KGaA, Weinheim.

Supplementary Information for:

IL-10 differentially controls the infiltration of inflammatory macrophages and antigen-presenting cells during inflammation.

Chia-Te Liao[1], Marcela Rosas[1], Luke C. Davies[1], Peter J. Giles[2], Victoria J. Hammond[1], Valerie B. O'Donnell[1], Nicholas Topley[3], Ian R. Humphreys[1], Donald J. Fraser[1], Simon A. Jones[1] and Philip R. Taylor[1]

Supplementary Methods

Adoptive Transfer Experiments

For adoptive transfer experiments, the peritoneal cells of naïve 129S6/SvEv.CD45.1 were recovered by lavage with 1 ml ice-cold PBS. Cells were pooled from several donors and 400 μl (1.8x10^6 cells) was intraperitoneally injected into 129S6/SvEv mice. One day later, mice were either injected with SES or left unchallenged. Total peritoneal cells were recovered after sacrifice 3 days later and analyzed by flow cytometry (see below).

Microarray analysis

CEL files were downloaded from GEO (GSE15907) before expression summarization using the RMA algorithm [1] in R. Differentially expressed genes were identified using limma [2] with P values corrected for multiple testing and false discovery [3].

Supplementary Tables

Table S1. Differentially expressed genes between F4/80^{low}MHCII^{+} and F4/80^{int}MHCII^{+} peritoneal cells during thioglycollate peritonitis [4]. Only genes showing a >5-fold difference in expression are shown. Note that Ccr2 exhibited a 3.17 fold increase on F4/80^{low}MHCII^{+} over inflammatory F4/80^{int}MHCII^{+} peritoneal cells. Abs FC, absolute fold change.

Probe ID	*P*-value	Abs FC	Symbol	Description
10576784	*1.29E-08*	*19.56224*	*Cd209a*	*CD209a antigen*
10483046	1.29E-08	18.37917	Dpp4	dipeptidylpeptidase 4
10457168	*2.14E-08*	*14.52031*	*Cd226*	*CD226 antigen*
10414262	2.07E-08	13.73705	Ear2	eosinophil-associated, ribonuclease A family, member 2
10419154	1.26E-07	12.46663	Ear1	eosinophil-associated, ribonuclease A family, member 1
10541581	5.55E-08	11.95879	Clec4b1	C-type lectin domain family 4, member b1
10419156	2.14E-08	11.52477	Ear10	eosinophil-associated, ribonuclease A family, member
10576829	1.25E-08	11.52477	Cd209c	CD209c antigen
10598175	1.31E-08	11.26155	Ear10	eosinophil-associated, ribonuclease A family, member
10538791	1.13E-07	10.0329	Tnip3	TNFAIP3 interacting protein 3
10415392	2.71E-08	9.781122	Ltb4r1	leukotriene B4 receptor 1
10399691	8.32E-09	9.042155	Id2	inhibitor of DNA binding 2
10497345	3.02E-08	8.574188	751864	predicted gene, 751864
10351644	9.29E-07	7.835362	Cd244	CD244 natural killer cell receptor 2B4
10548552	1.54E-07	7.799239	Klra2	killer cell lectin-like receptor, subfamily A, member 2
10548314	2.69E-06	7.464264	Klrb1b	killer cell lectin-like receptor subfamily B member 1B
10352000	5.36E-07	7.143676	Kmo	kynurenine 3-monooxygenase (kynurenine
10538802	4.07E-08	6.868523	A930038C07Rik	RIKEN cDNA A930038C07 gene
10538356	2.07E-08	6.528116	Chn2	chimerin (chimaerin) 2
10362896	1.54E-07	6.483023	Cd24a	CD24a antigen
10597743	3.13E-08	6.379014	Cx3cr1	chemokine (C-X3-C) receptor 1
10548105	4.94E-07	6.276673	Ccnd2	cyclin D2
10364038	4.73E-08	6.247735	Upb1	ureidopropionase, beta
10499189	1.53E-08	6.218931	Fcrls	Fc receptor-like S, scavenger receptor
10548345	1.98E-06	6.090947	Klrk1	killer cell lectin-like receptor subfamily K, member 1
10555297	2.48E-08	5.656854	Kcne3	potassium voltage-gated channel, Isk-related subfamily,
10530827	1.49E-07	5.451551	Spink2	serine peptidase inhibitor, Kazal type 2
10538150	6.04E-07	5.36409	Tmem176a	transmembrane protein 176A
10439542	2.31E-06	5.278032	Zdhhc23	zinc finger, DHHC domain containing 23
10470529	3.44E-07	5.074738	Olfm1	olfactomedin 1
10569646	2.71E-08	5.063026	Ccnd1	cyclin D1
10351792	1.89E-06	5.039684	Slamf9	SLAM family member 9

Table S2. Antibodies used in this study were purchased from BD Biosciences, Biolegend, eBioscience, AbD Serotec or R & D Systems. Biotin-conjugated antibodies were detected with PE-TexasRed-, allophycocyanin-, Peridinin-chlorophyll-protein complex (PerCP)- or Alexa Fluor 405-conjugated streptavidin.

Antibody	Clone
anti-F4/80-phycoerythrin (PE)-TexasRed	BM8
anti-F4/80-Pacific Blue/Biotin	CI:A3-1
anti-CD11b-allophycocyanin-Cy7/FITC	5C6
anti-CD11c-PE-Cy7	HL3
anti-CD19-V450	1D3
anti-CD45.1-allophycocyanin	A20
anti-CD45.2-allophycocyanin-Cy7	104
anti-CD80-biotin	16-10A1
anti-CD86-biotin	B7-2
anti-CD64 (FcγRI)-phycoerythrin (PE)	X54-5/7.1
Anti-CD103-allophycocyanin	M290
anti-Mer-allophycocyanin	108928
anti-CD192/CCR2-allophycocyanin	475301
anti-CD209a-biotin	5H10/CIRE
anti-CD226-PE/allophycocyanin/Alexa Fluor 647	10E5
anti-Ly6G-allophycocyanin-Cy7	1A8
anti-MHC-II I-A/I-E-PerCP/PE-Cy5.5	M5/114.15.2
anti-Tim4-PE	RMT4-54
anti-TNF-α-PE	MP6-XT22
anti-IL-6-PE	MP5-20F3
anti-IL-10-allophycocyanin	JES5-2A5
anti-IL-12p40/p70-PE	C15.6
anti-CD206-biotin	5D3

Supplementary Figures

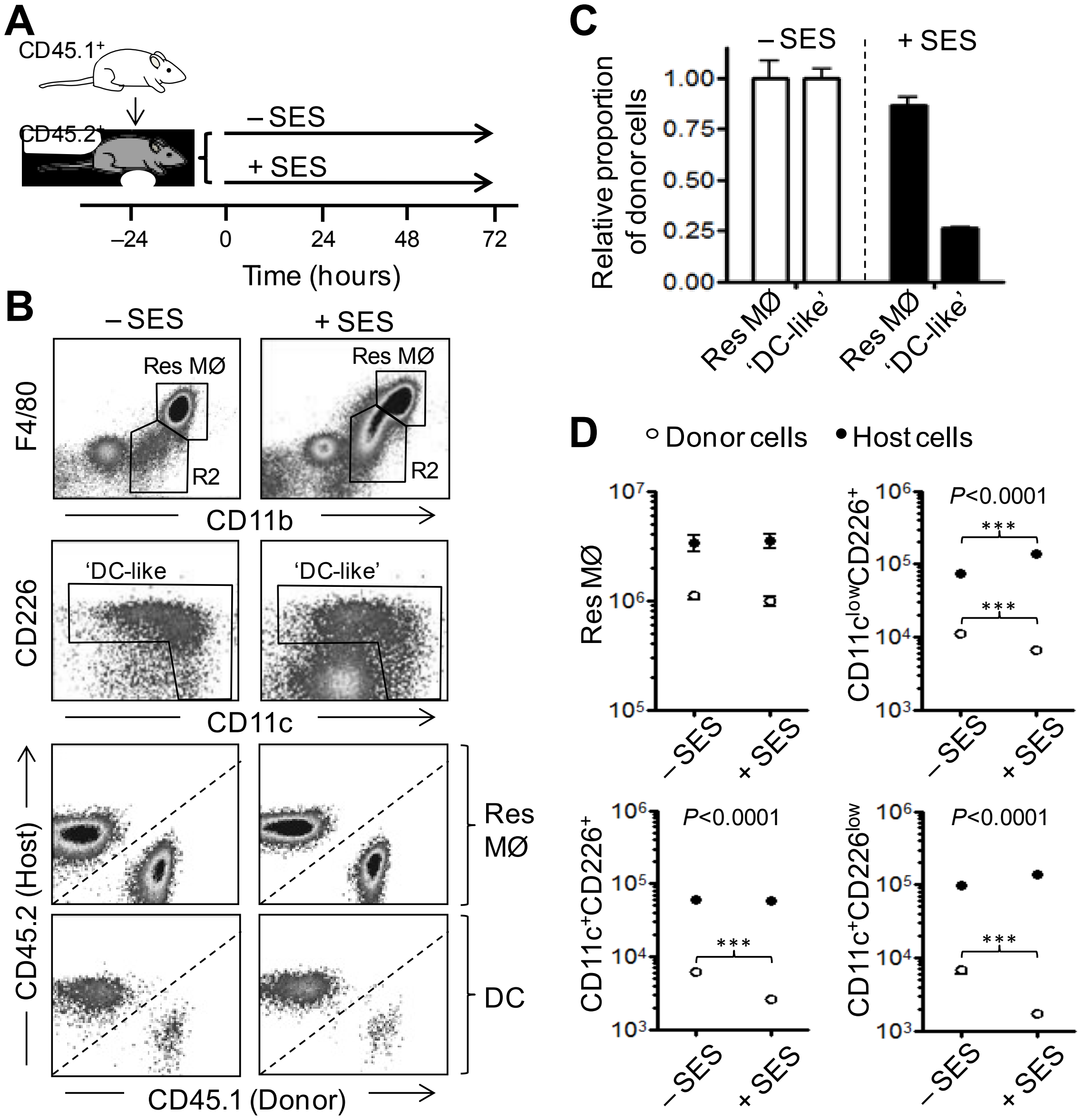

Figure S1. Peritoneal APC are recruited from periphery during peritonitis. A)

Schematic representation of the adoptive transfer strategy used to study to determine the origin of peritoneal APC during peritonitis. One day after adoptive transfer of naïve peritoneal cells between CD45 allotype-mismatched animals, mice were either injected intraperitoneally with SES or were left unchallenged and sacrificed on Day 3. **B**)

Representative flow-cytometric plots showing the relative contribution of donor and host cells to the ResMØ and APC populations 4 days after adoptive transfer. **'**R2' represents $CD11b^{int}F4/80^{int/low}$ population, which further subdivided into three subsets of APC and InfMØ based on CD11c and CD226 expression. Data are derived from 1 of 4 individual 129S6/SvEv mice. **C**) Bar graphs showing the relative proportion of $CD45.1^{+}$ donor cells in the peritoneal cavity of unchallenged and SES-challenged $CD45.2^{+}$ host mice 4 days after intraperitoneal adoptive transfer (normalized to unchallenged mice). Data represents the mean±SEM and are derived from 4 individual 129S6/SvEv mice. **D**) Graphs showing the number of recoverable donor (white symbols) and host (black symbols) ResMØ and APC in the peritoneal lavage of the mice shown in (C) above. Data represents mean±SEM. Data was analyzed for statistical significance by Two-way ANOVA after log transformation and interaction statistic P values and Bonferroni post-tests (asterisks) are indicated where significant.

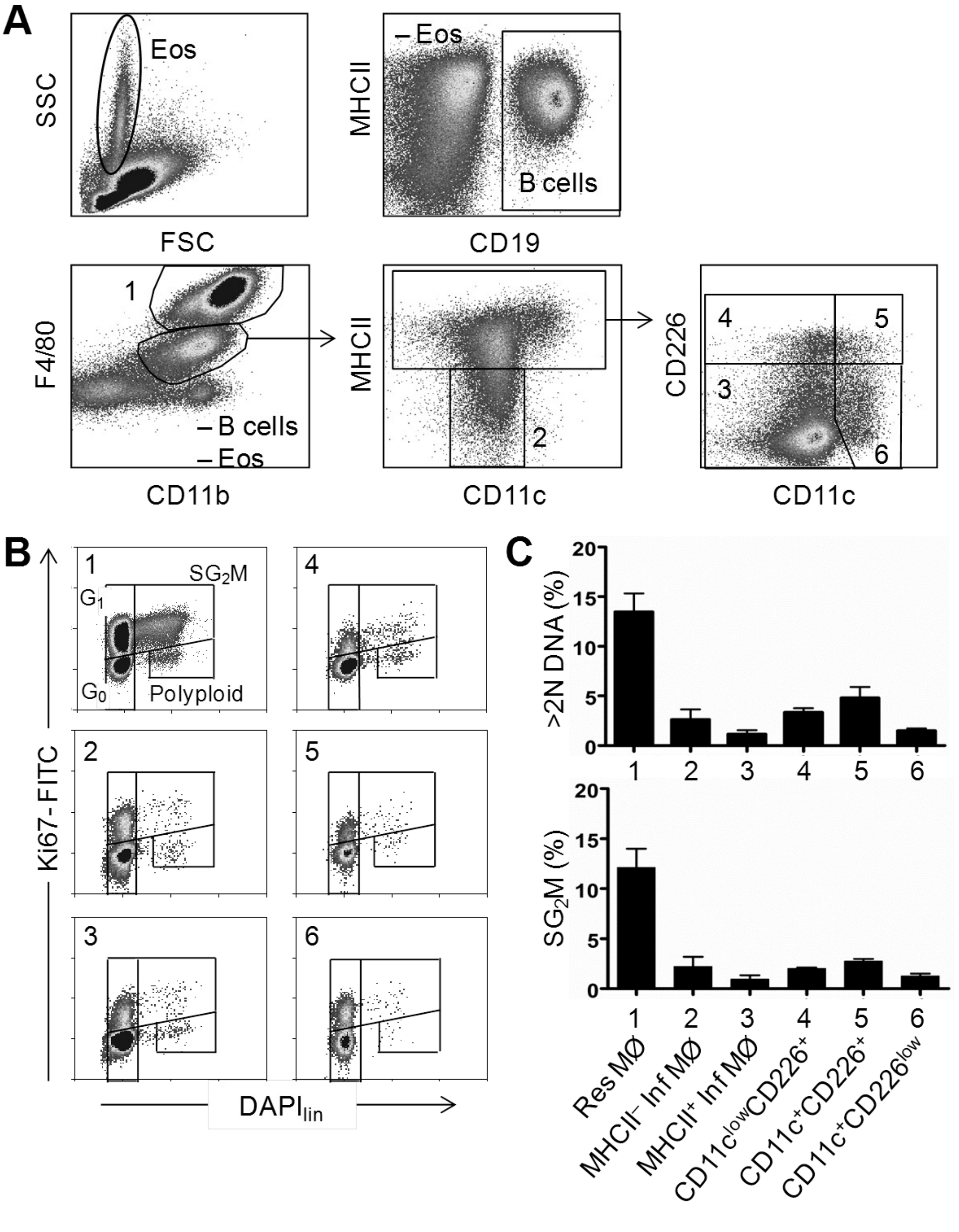

Figure S2. Limited evidence for proliferation of murine peritoneal APC during peritonitis. A) Peritoneal cells from mice 3 days after zymosan-induced peritonitis ($2x10^6$ particles) were analysed by flow-cytometry for evidence of cell cycle. Peritoneal MØ and myeloid APC were gated by exclusion of eosinophils (upper left panel) and $CD19^+$ B cells

(upper right panel), before selectively gating on F4/80^{high} ResMØ (gate 1, lower left panel) and F4/80$^{+/low}$ InfMØ and APC, which were further divided into MHCII^{-} InfMØ (gate 2, lower middle panel) and MHCII^{+} cells. The MHCII^{+} cells (lower middle panel) were further divided into MHCII^{+}CD226^{-}CD11c^{-} InfMØ (gate 3, lower right panel), and the 3 previously described APC populations (gates 4-6, lower right panel). **B**) The cells were assessed for active cell division during peritonitis as previously described [5] by measuring Ki67 expression and DNA content. **C**) Quantification of the data indicated that whilst ResMØ were experiencing substantial proliferation as previously observed, there was only limited evidence of proliferation in the APC subsets indicating that recruitment from the periphery may be the major source of these cells during peritonitis. Data represents the mean±SEM of 3 C57BL/6 mice from one of 2 similar experiments.

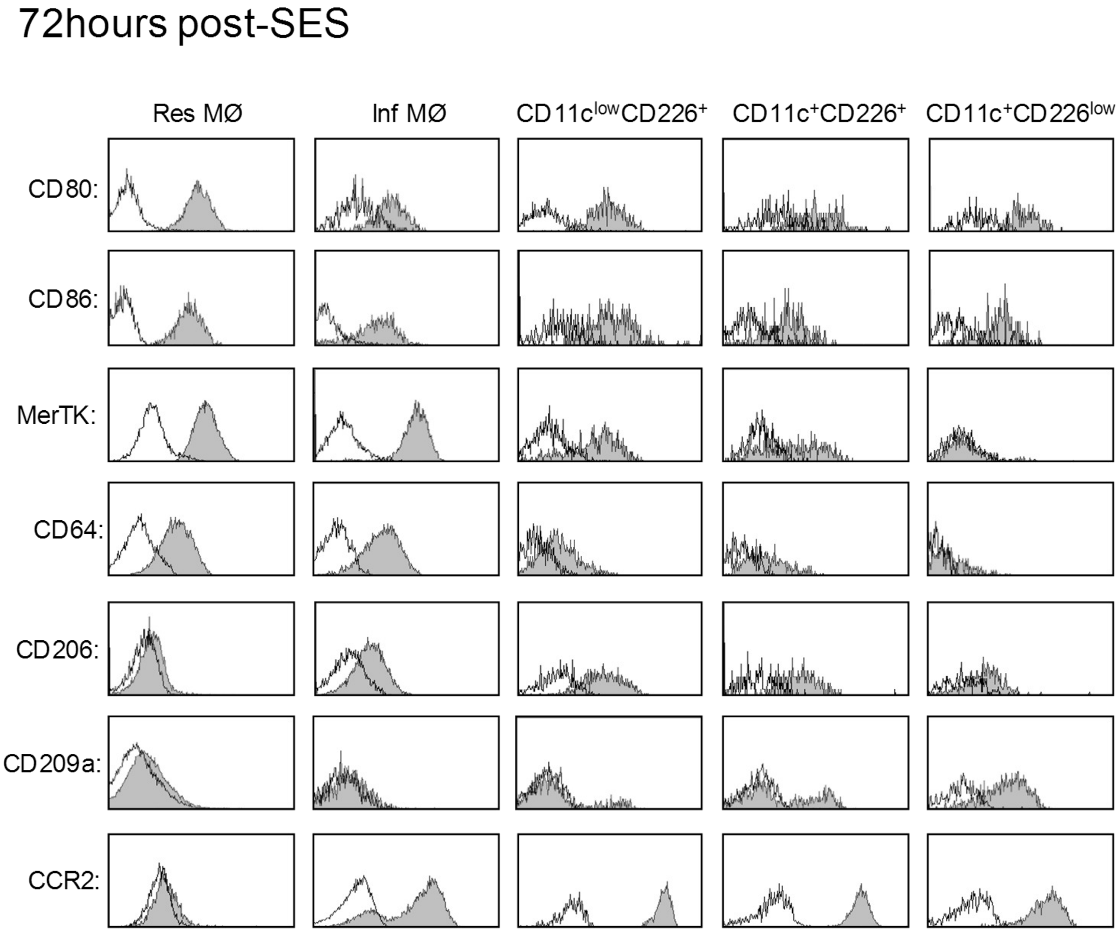

Figure S3. Phenotypic comparisons between peritoneal resident MØs, inflammatory MØs and three putative APC subsets from inflamed mice. Flow-cytometric analysis of select marker expression by murine peritoneal ResMØ, InfMØ and three 'DC-like' subsets. Representative histogram plots were pre-gated on respective MØ/DC subsets based on definitive phenotypic markers after exclusion of doublets, debris, $CD19^+$ B cells and $Ly6G^+$ neutrophils. Shaded histograms depict receptor specific staining and bold lines denote isotype control staining. Data are representative of two independent experiments with a total of four C57BL/6 mice.

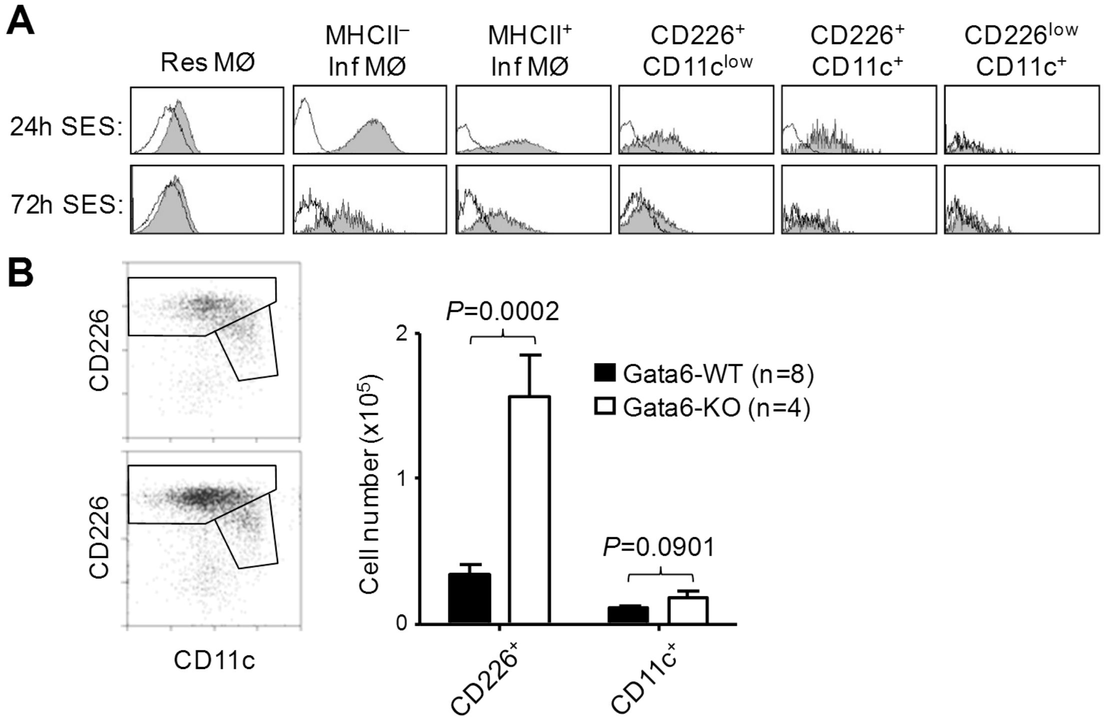

Figure S4. Two major subset of peritoneal APC. A) Analysis of Ly-6C expression on peritoneal myeloid cells during inflammation induced by intraperitoneal SES challenge indicated a transient expression most evident on the CD226 high cells, which could be consistent with monocytic origins. Shaded histograms and lies denote receptor-specific and isotype control staining. **B**) The appearance of two major populations with heterogeneous CD11c expression was supported by the observation that myeloid Gata6-deficient mice, which are known to exhibit an expansion in $MHCII^{high}$ APC like cells, had a selective enrichment of $CD226^{high}$ cells.

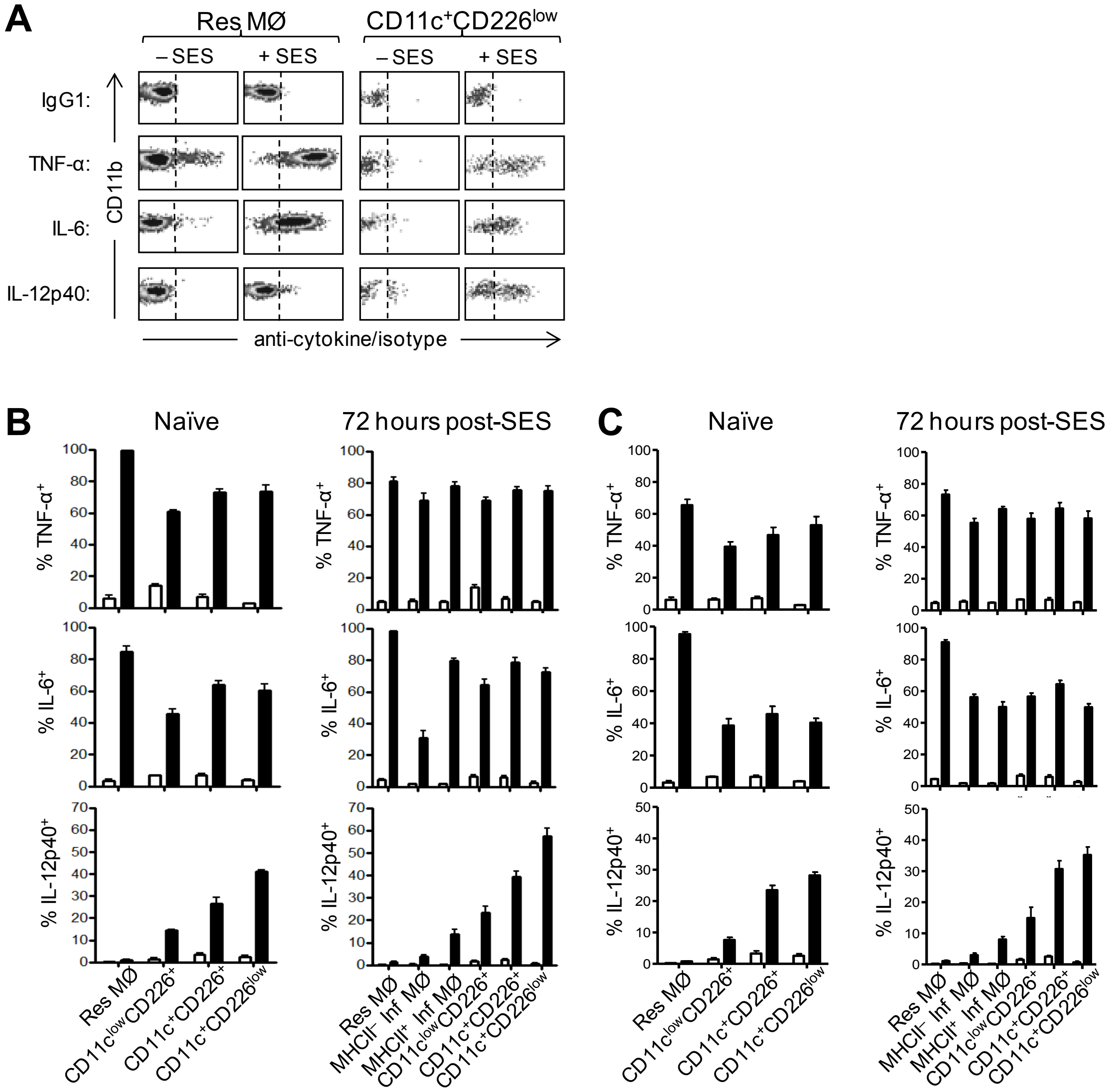

Figure S5. Murine peritoneal myeloid cells exhibit differential cytokine production following microbial products. A) Representative density plots depicting the flow-cytometric determination of intracellular cytokine production by peritoneal MØ and DC from naive C57BL/6 mice, after ex vivo stimulation with SES for 6 hours. **B**) Quantification of the intracellular cytokine production by subsets of peritoneal MØ and DC from C57BL/6 mice, both naïve (left) and 72 hours post-intraperitoneal in vivo SES challenge (right). Cells were

stimulated ex vivo with SES (black) or cultured in medium alone (white). Positive intracellular cytokine production was determined by comparison with isotype controls. Data represent mean±SEM and are derived from one of two similar experiments (n = 4-5 in each experiment). C) Similar data were acquired when 100ng/ml of LPS was used instead of SES. Data represent mean±SEM and are derived from one of two similar experiments (n = 4-5 in each experiment).

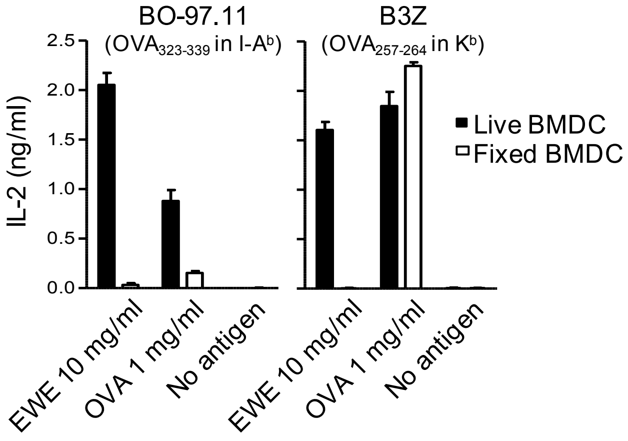

Figure S6. Validation of an Egg White Extract (EWE) for the assessment of processing of native antigen by peritoneal APC. EWE was compared to a commercial preparation of OVA in antigen processing and presentation assays. The ability of BMDC (both live and paraformaldehyde fixed) to process and present both sources of OVA to BO-97.11 and B3Z cells as a measure of presentation on MHCII and MHCI, respectively. The fixed BMDC were able to present commercial OVA, particularly to the B3Z cells, indicating the presence of degraded protein and peptides and hence not an effective measure of antigen processing. The EWE, however, was only presented to either reporter cell when the BMDC were live, indicating that antigen processing was required.

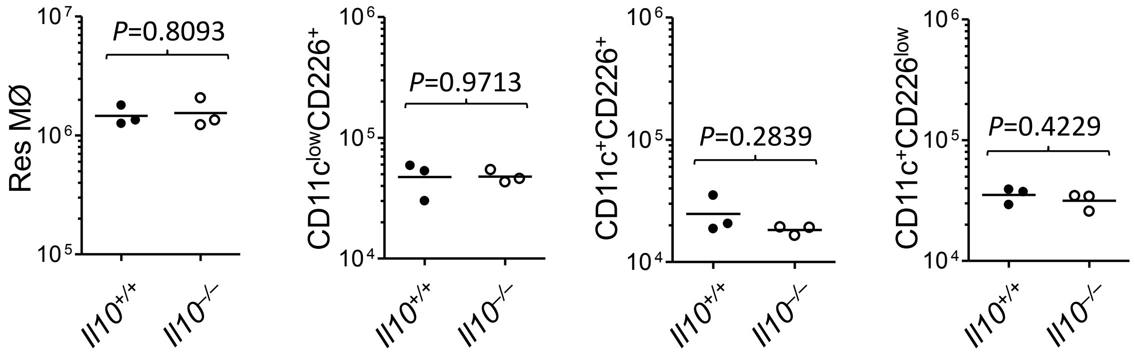

Figure S7. Peritoneal myeloid cells in naïve IL-10-deficient mice. IL-10-deficient mice have similar numbers of the peritoneal APC subsets and ResMØ to wild type mice. Horizontal lines denote means, data were analyzed by Student's t-test for the presence of statistically significant differences.

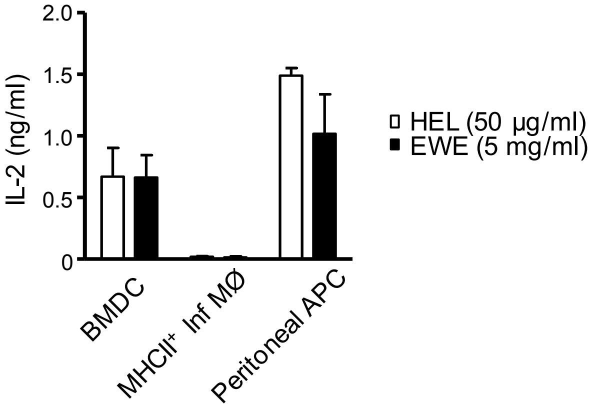

Figure S8. Processing and presentation of Hen Egg Lysozyme validates the differences between inflammatory MHCII$^+$ MØ and peritoneal APC. The ability of the MHCII$^+$ InfMØ to process and present EWE and hen egg lysozyme (HEL) on MHCII to 2G7.1 cells was assessed and compared to BMDC and peritoneal APC (both CD11c$^+$ and CD226$^+$ cells). Similar to the results with ovalbumin and BO-97.11 cells, the InfMØ were found to be poor at antigen processing and presentation. Data shows mean±SEM of IL-2 production by the 2G7.1 T cell hybridoma from triplicate samples and are representative of two independent experiments.

Supplementary References

1 **Irizarry, R. A., Bolstad, B. M., Collin, F., Cope, L. M., Hobbs, B. and Speed, T. P.,** Summaries of Affymetrix GeneChip probe level data. Nucleic Acids Res 2003. **31**: e15.

2 **Smyth, G. K.,** Limma: linear models for microarray data. In **Gentleman, R., Carey, V., Huber, W., Irizarry, R. A. and Dudoit, S.** (Eds.) Bioinformatics and computational biology solutions using R and Bioconductor. Springer, New York 2005, pp 397-420.

3 **Benjamini, Y. and Hochberg, Y.,** Controlling the false discovery rate: a practical and powerful approach to multiple testing. Journal of the Royal Statistical Society, Series B. 1995. **57**: 289-300.

4 **Gautier, E. L., Shay, T., Miller, J., Greter, M., Jakubzick, C., Ivanov, S., Helft, J., Chow, A., Elpek, K. G., Gordonov, S., Mazloom, A. R., Ma'ayan, A., Chua, W. J., Hansen, T. H., Turley, S. J., Merad, M., Randolph, G. J. and Immunological Genome, C.,** Gene-expression profiles and transcriptional regulatory pathways that underlie the identity and diversity of mouse tissue macrophages. Nat Immunol 2012. **13**: 1118-1128.

5 **Davies, L. C., Rosas, M., Smith, P. J., Fraser, D. J., Jones, S. A. and Taylor, P. R.,** A quantifiable proliferative burst of tissue macrophages restores homeostatic macrophage populations after acute inflammation. Eur J Immunol 2011. **41**: 2155-2164.
